# Humoral responses to the measles, mumps and rubella vaccine are impaired in Leigh Syndrome French Canadian patients

**DOI:** 10.1371/journal.pone.0239860

**Published:** 2020-10-21

**Authors:** Adrien Fois, Anne-Marie Boucher-Lafleur, Julie Thompson Legault, Christian Renaud, Charles Morin, Christine Des Rosiers, Lise Coderre, Catherine Laprise, Sylvie Lesage, LSFC Consortium

**Affiliations:** 1 Immunology-oncology Section, Maisonneuve-Rosemont Hospital Research Center, Montréal, Québec, Canada; 2 Département de microbiologie, infectiologie et immunologie, Université de Montréal, Montréal, Québec, Canada; 3 Centre intersectoriel en santé durable, Université du Québec à Chicoutimi, Saguenay, Québec, Canada; 4 Research Center, Montreal Heart Institute, Montréal, Québec, Canada; 5 Hôpital de Chicoutimi, Chicoutimi, Québec, Canada; University College London, UNITED KINGDOM

## Abstract

Leigh Syndrome French Canadian (LSFC) is a rare autosomal recessive metabolic disorder characterized by severe lactic acidosis crises and early mortality. LSFC patients carry mutations in the Leucine Rich Pentatricopeptide Repeat Containing (*LRPPRC*) gene, which lead to defects in the respiratory chain complexes and mitochondrial dysfunction. Mitochondrial respiration modulates cellular metabolic activity, which impacts many cell types including the differentiation and function of immune cells. Hence, we postulated that, in addition to neurological and metabolic disorders, LSFC patients may show impaired immune activity. To gain insight into the quality of the immune response in LSFC patients, we examined the response to the measles, mumps and rubella (MMR) vaccine by measuring antibody titers to MMR in the plasma. In a cohort of eight LSFC patients, the response to the MMR vaccine was variable, with some individuals showing antibodies to all three viruses, while others had antibodies to two or fewer viruses. These results suggest that the mutations in the LRPPRC gene present in LSFC patients may affect the immune response to vaccines. Monitoring vaccine response in this fragile population should be considered to ensure full protection against pathogens.

## Introduction

Leigh Syndrome is a neurodegenerative disease that results from genetic mutations leading to mitochondrial dysfunction [1,2]. A variant of this disease, Leigh Syndrome French Canadian (LSFC), was first described in the Saguenay-Lac-Saint-Jean region of Québec, where the prevalence is estimated to be 1 in 2000 births [3,4]. In addition to characteristic facial features, LSFC patients have reduced motor skills and show mental retardation [5] (MIM #220111). LSFC differs from the classical Leigh Syndrome in the occurrence of acute and unpredictable metabolic acidosis crises that significantly increase the severity and mortality of the disease [3–5]. Two causal mutations, namely A354V and C1277STOP, were identified in the nuclear Leucine Rich Pentatricopeptide Repeat Containing (*LRPPRC*) gene, which is involved in translating mitochondrial genes [6]. Mutations in this gene decrease the expression of *LRPPRC* causing a tissue-specific defect in respiratory chain complexes, affecting predominantly complex IV (cytochrome c oxidase) [7–9]. Other mutations in *LRPPRC* were more recently reported in Asia and Europe, resulting in similar phenotypes to LSFC [10–12]. This defect in the respiratory chain leads to multiple perturbations in energy nutrient metabolism beyond the commonly reported high blood lactate which could also contribute to some clinical manifestations observed in patients [12–14].

Cellular metabolism plays a crucial role in the function and differentiation of immune cells [15–17], such that mitochondrial dysfunction may induce alterations of the immune system that could negatively impact the health of patients [18]. Interestingly, the response to infections coincides with the development of metabolic acidosis crises in LSFC patients [5]. Preventing infections through vaccination may be an effective means to prevent crises. Yet, the efficacy of response to vaccines has not been tested in LSFC patients. As vaccination protocols solicit various components of the immune system to provide long-lasting protection against pathogens, measuring the response to vaccines will inform us on the quality of the immune response in LSFC patients. We selectively opted to study the response to the measles, mumps and rubella (MMR) vaccine for the following reasons: this trivalent vaccine is routinely administered in the province of Québec, it triggers a durable humoral immune response with a high rate of responders [19], there is a robust assay to quantify antibodies to MMR [20], and the MMR vaccine provides efficient protection to these three viruses [21–23]. Indeed, the protection from measles is at 85% to 95% after administration of the first vaccine and is greater than 95% after the second dose of the vaccine [24–26]. Protection from mumps and rubella after the administration of the first dose of MMR vaccine is typically at 62%-91% and 95%, respectively [24–26]. In contrast to measles, the protection only modestly improves upon secondary challenge for both mumps and rubella [24–26]. In Québec, the first immunization to measles has been typically administered within the trivalent MMR vaccine, whereas the second immunization was either MMR or measles alone. The vaccination schedule has recently changed, and now proposes two immunizations with the MMR vaccine in combination with varicella [27].

Protection against measles, mumps and rubella is of interest, as these viruses can cause severe pathologies [21–23]. Specifically, measles causes symptoms reminiscent of the common cold, including fever, runny nose, cough, watery eyes, and it is characterized by the appearance of white spots in the mouth, followed by the appearance of a characteristic rash [21]. In some cases, however, measles is also associated with severe complications that can lead to blindness, encephalitis, diarrhea, pneumonia and causes more than 100 000 deaths yearly, worldwide [21]. Mumps is less severe than measles and can cause severe swelling of the parotid glands, the pancreas, the testicles, as well as infect the central nervous system occasionally leading to death [22]. Finally, rubella is usually benign with most infected individuals presenting with no or few symptoms, such as a rash and a mild fever [23]. However, in pregnant women, rubella can trigger miscarriages or cause fetus malformations [23]. All of these pathologies are preventable through vaccines that lead to a robust humoral immune response quantifiable by the presence of serum antibodies [21–23].

To examine the quality of the humoral response in LSFC patients, we analyzed plasma samples that had been previously collected as part of a study investigating the metabolic signatures in the plasma of LSFC patients [13,14]. We observed that some LSFC patients can mount a detectable antibody response to MMR. However, the overall response rate is relatively lower to that reported in the general population. Altogether, these results show that MMR vaccination is sufficient to induce antibody responses in some LSFC patients, while the humoral immune response to MMR in other LSFC patients seems to be somewhat impaired.

## Materials and methods

### Subjects

Recruitment of LSFC patients and control subjects occurred between September 2011 and April 2012 [13,14]. LSFC patients and control subjects were age-matched. Control subjects were selected based on the following exclusion criteria: positive genotyping result for A354V or C1277STOP mutations of the LRPPRC gene, smoking, and known diseases (ex: diabetes, cancer, other mitochondrial disorders, etc.). Vaccination history was obtained by asking either the participants or their legal guardian to provide the information included in the vaccination booklet, or by asking their physician to extract the information from their medical records, as per the methodology used to report vaccine coverage in Canadian children [28,29].

### Ethics approval and consent to participate

Blood samples were obtained from all participants after receiving written informed consent from either the participant or their legal guardian. The protocol was approved by the Research Ethics Committees of the Centre intégré universitaire de santé et de services sociaux (CIUSSS) de Saguenay–Lac-Saint-Jean and Université du Québec à Chicoutimi. All methods were carried out in accordance with relevant guidelines and regulations of the CIUSSS de Saguenay-Lac-Saint-Jean and Université du Québec à Chicoutimi.

### MMR vaccine

The MMR vaccine is composed of three attenuated live viruses, including measles, mumps and rubella. It causes a harmless infection, with very low to no symptoms, and effectively triggers the immune system to generate immune memory [21–23]. In Quebec, the first dose of this vaccine is scheduled to be administered at 12 months, and the second dose at 18 months [28]. For the participants included in this study, this second dose could contain measles only [28], as their vaccination precedes the application of the recent changes to the immunization schedule [27]. All participants from whom we obtained vaccination information received the first dose of MMR between the ages of 12 to 15 months. The type of vaccine (MMR or measles only) and the age of the individuals at the second dose are noted in Tables 1 and 2, when the information was available. No adverse events to vaccination were reported in the accessible medical information, by the subject themselves, by their legal guardian nor by their physician.

**Table 1:** Vaccine information and antibody titers for control subjects.

		First vaccine		Second vaccine			Plasma antibody titers and interpretation (+ or -)					
*Subject ID*	Sex	Type	Age (months)	Type	Age (years)	Age of patient at collection (years)	Measle IgG (AU/ml)		Mumps IgG (AU/ml)		Rubella IgG (IU/ml)	
*337*	M	MMR	12	Measles	15	30	99.8	+	30.8	+	33.3	+
338*	F	MMR	15	Measles	17	32	163	+	226	+	10	+
*339*	F	N/A	-	N/A	-	12	16	+	14.5	+	46.5	+
*340*	M	N/A	-	N/A	-	13	>300	+	58.4	+	26.4	+
*341*	F	MMR	14	Measles	9	24	6.15	-	77.7	+	28.9	+
*342*	F	None	-	None	-	25	<5.0	-	<5.0	-	0.1	-
*343*	F	MMR	12	MMR	2	8	>300	+	56.4	+	30.4	+
*346*	M	N/A	-	N/A	-	27	72.5	+	113	+	50.7	+

* Double-dose.

N/A information not available.

**Table 2:** Vaccine information for LSFC patients.

		First vaccine		Second Vaccine		
*Patient ID*	Sex	Type	Age (months)	Type	Age (years)	Age of patient at sample collection (years)
101*	M	N/A	N/A	N/A	N/A	30
*102*	M	N/A	N/A	N/A	N/A	30
*103*	F	MMR	12	No	-	28
*104*	M	MMR	13	MMR	2	11
*105*	F	MMR	13	Measles	3	22
*106*	F	No	-	No	-	13
*108*	F	N/A	N/A	N/A	N/A	10
*110*	F	MMR	12	No	-	22

* Compound heterozygous patient.

N/A information not available.

### Blood collection and serology testing

Samples were collected following a standardized protocol to minimize variability related to environmental factors [14]. Blood samples were drawn in EDTA BD vacutainer tubes, directly placed on ice and centrifuged within 30 min of collection at 2 000 x *g* for 15 min. Plasma was aliquoted into microtubes, frozen on dry ice and stored at -80°C until analysis. The serology for measles IgG, mumps IgG and rubella IgG were performed by a clinical diagnostic laboratory using CMIA technique on the Liaison XL, Diasorin (www.diasorin.com). For measles, the assay range is 5–300 AU/ml, with diagnostic specificity and sensitivity of 97.4% and 94.7%, respectively. For mumps, the assay range is 5–300 AU/ml, with diagnostic specificity and sensitivity of 98.2% and 98.5%, respectively. For rubella, the assay range is 0.2–350 IU/ml, with diagnostic specificity and sensitivity of 100% in vaccinated individuals.

### Statistics

Differences in antibody titer data from control subjects and LSFC patients were tested for significance using a nonparametric two-tailed Mann-Whitney U test. A one proportion binomial test was applied to determine if the proportion of responders differs from the expected value. The minimal significance threshold was set at 0.05 for all tests.

## Results

In the province of Québec, there are currently eleven individuals diagnosed with LSFC. Due to their precarious clinical conditions, it is difficult to obtain blood samples from these individuals. Therefore, we opted to exploit LSFC patient plasma samples that were collected as part of a previous study, which aimed at characterizing the metabolic signature of these patients [13,14]. Although nine patients were initially included, plasma was available only for eight subjects. Among the eight LSFC patients, seven are homozygous for the A354V mutation and one patient is a compound heterozygote for the A354V and C1277STOP mutations [6]. The LSFC patient plasma samples were collected in 2011 and 2012 and were carefully stored at -80°C.

Both the time of collection and the storage could affect our ability to detect antibodies to MMR. To verify sample quality, we analyzed blood samples from age-matched control subjects that were collected at the same time as those from the LSFC patients. Because the previous study was not initially designed to look at immune responses, vaccination information for the participants was collected retrospectively, and is thus incomplete. Still, vaccination information was available for five of the control subjects, one of whom was not vaccinated (Table 1). The four vaccinated control subjects had received the first MMR vaccine at about one year of age (Table 1). Subject 338, marked with an asterisk, received a double dose of MMR at her first immunization. Subject 343 received a second MMR vaccine at two years of age, while the other three received a measles only vaccine, at 9, 15 and 17 years of age. Using the plasma samples from all control subjects, we quantified the antibody titers to measles, mumps and rubella. The antibody titer detection threshold is set at 10 AU/ml for measles and mumps, and 10 IU/ml for rubella [30]. Unsurprisingly, the antibody titer for the unvaccinated individual 342 was well below the detection threshold (Table 1). In contrast, all four control subjects with a confirmed MMR vaccination status were seropositive for antibodies to mumps and rubella, whereas all but subject 341 were antibody seropositive for measles (Table 1). The measles antibody seronegative subject 341 may fall within the reported 5% individuals that do not respond to the measles arm of the vaccine [24–26]. The three control subjects for whom vaccination information could not be retrieved were also seropositive for antibodies to the three viruses (Table 1). In Québec, the low prevalence of these diseases as well as the high rate of compliance to vaccination suggest that these triple antibody seropositive individuals have been vaccinated [28]. Altogether, these data show that our plasma sample collection and storage were adequate, as plasma samples from seven control subjects collected and stored in the same conditions as those from LSFC patients showed a typical response to the MMR vaccine.

We next turned our attention to plasma samples from LFSC patients. Of the eight LSFC patients, we obtained vaccination information for five patients, one of whom was not vaccinated (Table 2). In accordance with the recommended provincial guidelines, the four vaccinated LSFC patients received the first dose of the MMR vaccine at about one year of age (Table 2). A second vaccine dose was only confirmed for LSFC patients 104 and 105, receiving MMR at two years of age and a measles only vaccine at three years of age, respectively. LSFC patients 103 and 110 did not receive a second immunization (Table 2). We quantified antibody titers of the eight LSFC patients. Because the antibody seroconversion rate following vaccination is different for measles, mumps, and rubella, we discuss the antibody seroconversion for each virus, individually.

**Fig 1:**
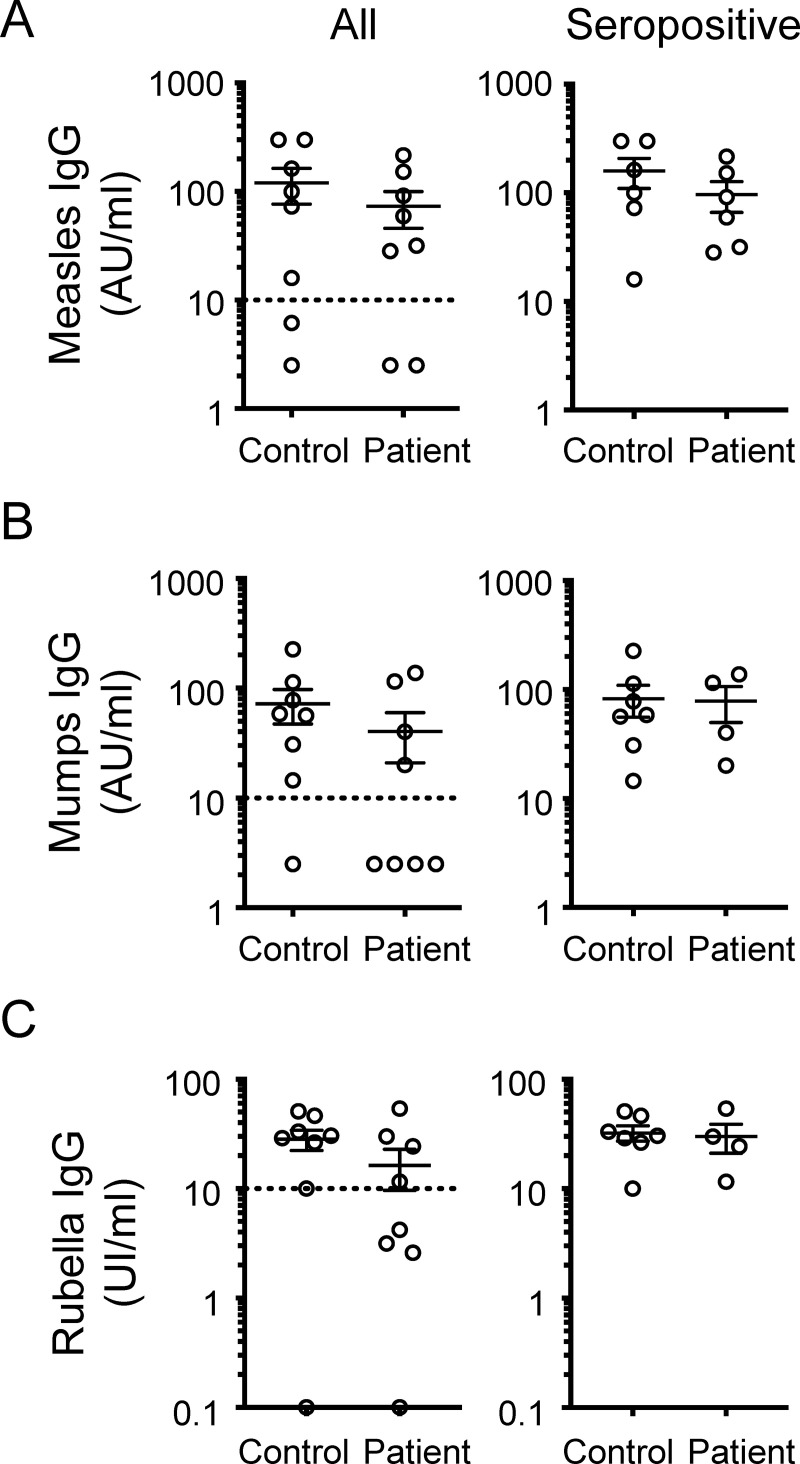
Antibody titers in seropositive LSFC patients are comparable to that of control subjects. IgG antibody titers for a) measles, b) mumps and c) rubella are plotted for all (left) and for seropositive (right) control subjects and LSFC patients. Each dot represents data for a single participant. Mean ± SEM values are shown. No significant differences were observed when comparing the antibody titer data from seropositive control subjects and seropositive LSFC patients using a nonparametric two-tailed Mann-Whitney U test, with *P* values > 0.05.

We first examined the plasma antibody levels to measles. As mentioned, not all LSFC patients received two doses of MMR and some vaccination information is missing (Table 2). Therefore, for comparison, we conservatively considered the expected seroconversion rate of a single dose of the vaccine, which is typically of 95% in the general population [24–26]. Predictably, for patient 106 which was not vaccinated, antibody levels to measles were below the detection limit (Table 3). Among the seven other patients, all were seropositive for antibodies to measles, except patient 110 (Table 3). By excluding LSFC patient 106 that was not vaccinated, we obtain a response rate of 6 out of 7, or 86% (Table 4). The antibody seronegative LSFC patient 110 received a single dose of the MMR vaccine and may fall within the reported 5% of individuals that do not seroconvert following a single dose of the measles vaccine [24–26]. The seroconversion rate in this small sample size is thus not much different than that of the general population (Table 4). In addition to the seroconversion rate, we also quantified the plasma antibody titers. In Fig 1A, we plot the serology data from the all control subjects and LSFC patients, including the two participants that were not vaccinated. As noted above, two control subjects and two LSFC patients had antibody levels below the detection limit (Fig 1A). The measles antibody titers from the seropositive LSFC patients were comparable to those from the seropositive control subjects included in our study (Fig 1A). Together, the response rate and antibody titers suggest that LSFC patients mount a humoral response to measles after the MMR vaccine.

**Table 3:** Antibody titers and seroconversion interpretation for LSFC patients.

*Patient ID*	Vaccine doses	Measles IgG (AU/ml)		Mumps IgG (AU/ml)		Rubella IgG (IU/ml)		Global response to MMR
101*	N/A	91.4	+	<5.00	-	3.15	-	unconfirmed
*102*	N/A	28.2	+	40.1	+	24.4	+	responder
*103*	1	59.4	+	<5.00	-	2.6	-	non-responder
*104*	2	31.8	+	115	+	11.6	+	responder
*105*	2	152	+	138	+	30	+	responder
*106*	0	<5.0	-	<5.0	-	0.1	-	-
*108*	N/A	216	+	20	+	54	+	responder
*110*	1	<5.0	-	<5.0	-	4.2	-	non-responder

* Compound heterozygous patient.

N/A information not available.

**Table 4:** Antibody seroconversion in LSFC patients based on a single dose administration of the MMR vaccine.

	Expected seroconversion	Observed seroconversion	Seroconversion without patient 101
***Measles***	95%	86% (6/7)	83% (5/6)
***Mumps***	95%	57% (4/7)*	67% (4/6)*
***Rubella***	97%	57% (4/7)*	67% (4/6)*
***Global MMR***	95%	57% (4/7)*	67% (4/6)*

* *P* value < 0.05.

Second, we considered the antibody seroconversion to mumps. In the general population, it is estimated that the first dose of the MMR vaccine confers protection to mumps in 62 to 91% of individuals, even if the antibody seroconversion rate is of 95% [26]. In contrast to control subjects (Table 1), the antibody seroconversion to mumps was low in LSFC patients, with antibodies detected in only four of the eight LSFC patients (Table 3). The four mumps antibody seronegative patients include the unvaccinated patient 106. Still, we were able to confirm that two of the three other mumps antibody seronegative patients, namely patients 103 and 110, had received one dose of the MMR vaccine. The fourth mumps antibody seronegative LSFC patient, namely patient 101, had antibodies to measles, suggesting that he may have been vaccinated with MMR or exposed to measles. Overall, by excluding the unvaccinated patient, the antibody seroconversion to mumps is of 4/7 (57%). If we also exclude patient 101 for whom we could not confirm the vaccination status, the antibody seroconversion is 4 out of 6, or 67% (Tables 3 and 4). In this small cohort, a 67% antibody seroconversion to mumps is below the expected range of 95% after a single dose of the MMR vaccine (Table 4). Interestingly, as for measles, the plasma levels of antibodies to mumps in seropositive LSFC patients were comparable to those of antibody seropositive control subjects (Fig 1B). Altogether, these data suggest that the antibody seroconversion to mumps is diminished in some LSFC patients relative to the general population. Still, among those that seroconvert, the plasma antibody levels are within the expected range, suggesting that at least some LSFC patients exhibit a normal humoral response to mumps.

We lastly examined the response to rubella. After a single MMR vaccine, the antibody seroconversion to rubella is typically at 97% in the general population, conferring protection to 95% vaccinated individuals [24–26]. As for mumps, the rubella antibody seroconversion was very low in this small LSFC patient cohort. Antibodies to rubella were detected in only four patients (Table 3). Again, unvaccinated patient 106 did not have detectable levels of rubella antibodies and is not included in the calculation of seroconversion. When compared to the expected antibody seroconversion efficiency of 97% for rubella after a single vaccine dose, this low response rate of 4/7 (57%)–or of 4/6 (67%) if we exclude patient 101 for whom we could not confirm the vaccination status–suggests that the humoral immune response to rubella is ineffective in some LSFC patients (Table 4). Still, as for both measles and mumps, among the LSFC patients that showed rubella antibody seroconversion, the levels of antibodies were similar to that of control subjects (Fig 1C). This result suggests that some LSFC patients can respond to rubella as part of the MMR vaccine.

In addition to the specific response to each virus included in the MMR vaccine, we also aimed to determine the global clinical response rate to this vaccine among the LSFC patients. A clinical lack of response to the MMR vaccine is considered when the antibody titers to two out of three viruses are below the detection limit. In populations where the global MMR vaccination rate is near 90%, such as in the province of Québec [28], approximately 5% of individuals show seronegativity for two of the three viruses [31,32]. This proportion typically includes unvaccinated individuals. However, due to the small size of our LSFC patient cohort, we chose to exclude the data from the unvaccinated patient 106 to avoid undue negative bias. Within the seven other LSFC patients, four individuals (patients 102, 104, 105 and 108) had antibodies to all three viruses and are considered as responders (Table 3). Patients 103 and 110 were considered non-responders as they had received at least one dose of the MMR vaccine, yet were both seronegative for antibodies to two and three viruses, respectively (Table 3). Patient 101 was also seronegative for antibodies to mumps and rubella and, as discussed below, is likely to be a non-responder, establishing the response rate to 4 out of 7 LSFC patients (or 57%). Still, as we could not confirm the vaccination status of patient 101, we also excluded this patient from the calculation of the global clinical response rate to the MMR vaccine. By excluding both patients 101 (with unconfirmed vaccination status) and 106 (not vaccinated), the response rate of 4 out of 6 (or 67%) is below the expected response rate of 95% (Table 4). Altogether, these results suggest that although the humoral immune response to measles after MMR vaccine administration is within or near the expected response rate, the overall response to MMR vaccine of 57–67% in the small cohort of LFSC patients is lower than the expected 95% of the general population.

## Discussion

LSFC is an inherited mitochondrial disorder that alters cellular metabolism, which is known to influence the immune response. Yet there are few reports regarding the quality of the vaccine response in heritable mitochondrial disorders. Here, we quantified the humoral immune response to the MMR vaccine in LSFC patients. This vaccine is widely administered in the province of Québec and confers robust protection from three viruses, namely measles, mumps, and rubella. Vaccine responses in people with inherited mitochondrial disorder, such as LSFC, are currently not well understood and the response to vaccines can vary depending on the disease [33,34]. In our small cohort of LSFC patients, we find that the response to measles is likely comparable to that of control subjects, but the number of LSFC patients that developed antibodies to mumps and rubella is lower than expected. Moreover, the overall response rate to the MMR is also lower than that typically observed.

This study included plasma samples from eight LSFC patients. One of them was not vaccinated and we could not detect antibodies to measles, mumps or rubella in her plasma sample. Among the seven others, LSFC patients 103, 104, 105, and 110 received at least one dose of the MMR vaccine. Patients 102 and 108 had antibodies to all three viruses and were thus deemed responders. Indeed, due to the low prevalence of these diseases, it is unlikely that they would have been exposed to the three viruses, other than by receiving a vaccine. There is only patient 101 which was a non-responder (seronegative for two out of three antibodies to MMR) and for which we were unable to retrieve information regarding his vaccination status. He has developed antibodies to measles, but not to mumps and rubella. Although unlikely, we cannot exclude the possibility that this patient was not vaccinated and exposed to measles. As such, by excluding this latter patient, we calculated that the response to MMR was 4 out of 6 LSFC patients. Because we observed that at least four LSFC patients showed complete antibody responses to MMR, we believe vaccines inducing the humoral immune response, such as MMR, tetanus, hepatitis, polio, and influenza among others, will benefit this fragile patient group. Altogether, we propose that LSFC patients should be encouraged to receive adequate vaccination as well as to adhere to the standardized immunization protocols, especially when considering that infections in LSFC patients are associated with lactic acidosis crisis [2,3,5], a major cause of morbidity and death.

A recent study examined responses to multiple vaccines in patients with various other inherited mitochondrial disorders [35]. Globally, these patients showed considerably impaired responses to Haemophilus influenza type b, hepatitis B, and varicella [35]. Of relevance, the antibody seroconversion to measles and mumps were also lower than for normal healthy individuals, while that of rubella was within the normal range [35]. As some patients with inherited mitochondrial disorders are immunocompromised, the rate of antibody seroconversion to given vaccines may vary [33,34]. Stratifying the findings based on given inherited mitochondrial disorders, may provide information of the types of mutations that are most important in driving defects in humoral immune responses. In that regard, it would be of interest to determine the quality of the humoral immune response to other vaccines in LSFC patients.

In LSFC patients, the reason why the antibody seroconversion rate for both mumps and rubella is lower than expected, while that of measles is near the normal range is unclear. The type of immune response required to mount effective protection to these viruses may differ. Indeed, although measles, mumps and rubella are all single strand RNA viruses, they exhibit different properties [21–23]. For instance, measles and mumps are both part of the *Paramyxoviridae* family, whereas rubella is part of the *Togaviridae* family of viruses [21–23]. Each of these families of viruses bear distinct properties, such as differences in RNA polarity, capsid structure, virion size, and more, all of which could differentially influence the capacity of the host to mount an effective immune response [36–38]. For instance, while measles infection down-regulates signaling through the RIG-I-like pathway [39,40], effective immune responses to mumps is at least partially dependent on this innate immune sensor pathway [41]. Moreover, host genetic factors influence the efficacy of the response to rubella [23]. In the context of patients carrying a mutation in *LRPPRC*, these differences may further contribute to the distinct outcomes of the immune responses. As documented for hepatitis C, different viral proteins could also influence the efficacy of the immune response in LSFC patients by directly interacting with LRPPRC [42]. Alternatively, if not for the differences in the virus themselves, the reason for the lower antibody seropositivity to mumps and rubella could be due to differences in patient viral exposure. As recent measles outbreaks are more frequent than for mumps or rubella [21–23], seropositivity to measles may have increased in exposed individuals. Still, it should be noted that the health status of LSFC patients is closely monitored and infections to measles, mumps or rubella are not listed in the information available on their medical history, suggesting that LSFC patients are unlikely to have previously contracted these diseases. In addition, the compliance to the first dose of the MMR vaccine is high in the province of Québec [28,29], and outbreaks of measles, mumps or rubella are rare, such that exposure to these viruses in LSFC patients that live in somewhat isolated and protected conditions are unlikely. Therefore, although exposure to these viruses cannot be formally excluded, antibody seropositivity to measles, mumps or rubella in LSFC patients is most likely the result of vaccination. Additional studies are needed to clarify the reason why some LSFC patients show a poor response to both mumps and rubella.

Our study is limited by two major factors. First, the size of our cohort is small. As LSFC is a very rare disease, with a high mortality rate in early childhood, we had access to only eight samples from LSFC patients. A second limitation was the inability to retrospectively acquire the information regarding both the virus exposure and the vaccination protocol mostly due to out of date contact information or loss of information by the patient or their representative. Nevertheless, we were able to obtain sufficient information to conclude that some LSFC patients can mount a normal and protective immune response to the MMR vaccine.

The control subject cohort was limited by these same factors. As for LSFC patients, we only had access to eight plasma samples that were collected and stored in the same conditions as that of the LFSC patients. In addition, we were only able to retrieve vaccination information for five subjects, one of whom was not vaccinated. Still, the three control subjects for which the vaccination status was not confirmed were antibody seropositive for the three viruses, and were thus likely to have received at least one dose of the vaccine. Therefore, except for the unvaccinated subject, all control subjects had antibodies to at least two of three viruses, which suggest that they responded to MMR. This is in contrast to only four responders out of seven among the LSFC patients.

The number of LSFC patients that did not mount a quantifiable humoral immune response to MMR is greater than anticipated. These non-responders should be encouraged to obtain an additional dose of the MMR vaccine. It would be interesting to follow-up on both MMR vaccine responders and non-responders within the LSFC patients to determine whether the absence of response to MMR is generalizable to other vaccines, and to explore what drives this difference at the cellular level. Additional longitudinal studies monitoring vaccine response in patients with mitochondrial diseases may reveal that vaccination protocols need to be adapted to better suit this population. For instance, additional recall responses may prove to be necessary to provide more efficient protection. In that regard, the two LSFC patients that we know adhered to the recommended provincial guidelines, by receiving two doses of the vaccine in early childhood, showed antibody seroconversion to the three viruses.

In conclusion, we here quantified the humoral response to the MMR vaccine in eight LSFC patients by measuring antibody levels in the plasma. In this small cohort, the antibody seroconversion rate in response to MMR was lower than expected. Follow-up studies are needed to define whether the humoral response is generally impaired in LSFC patients. In the meantime, as antibody seroconversion in response to MMR was detected in some LSFC patients, that no adverse events were reported following MMR vaccination, and that, in some instances, acidotic crises coincide with infections, we feel that patients should be encouraged to adhere to current vaccination protocols to prevent them from developing more serious pathologies that may be significantly detrimental to their health.

## Abbreviations

LSFC: Leigh Syndrome French Canadian
LRPPRC: Leucine Rich Pentatricopeptide Repeat Containing
MMR: Measles, mumps and rubella
